# Genetic structure in neotropical birds with different tolerance to urbanization

**DOI:** 10.1038/s41598-022-09961-9

**Published:** 2022-04-11

**Authors:** Mauricio Rodríguez-Bardía, Eric J. Fuchs, Gilbert Barrantes, Ruth Madrigal-Brenes, Luis Sandoval

**Affiliations:** 1 Programa de Posgrado en Biología, Sistema de Estudios de Posgrado, Universidad de Costa Rica. San Pedro, San José, 11501-2060 Costa Rica; 2 Escuela de Biología, Universidad de Costa Rica. San Pedro, San José, 11501-2060 Costa Rica

**Keywords:** Population genetics, Zoology, Tropical ecology, Urban ecology

## Abstract

Gene flow in birds can be affected by urbanization depending on natural history traits and adaptability to habitat change. Contrasting results can be expected when comparing species with opposite resilience to urbanization. In this study, we assessed genetic diversity and structure for two bird species, the urban avoider white-eared ground-sparrow, *Melozone leucotis,* and the urban dweller house wren *Troglodytes aedon*. We used seven microsatellite loci and sampled five locations with differing levels of urbanization in Costa Rica. We found considerably higher genetic structure in white-eared ground-sparrows than in house wrens. Circuit theory analyses proved a higher isolation from urban resistance for the white-eared ground-sparrow than for house wrens. These results support that urbanization is a significant barrier for gene flow in urban avoiders, in contrast to urban dweller species that showed little to no impact. Differences could be attributed to a higher plasticity in habitat and nesting site preferences in the house wren, and significant dispersal limitation for the white-eared ground-sparrow. These results emphasize the need for conservation strategies towards white-eared ground-sparrows and other urban avoider species whose habitat and connectivity have been reduced by the recent urban expansion.

## Introduction

The rapid expansion of urbanized areas has converted large extensions of natural habitats into a concrete jungle that surrounds some small natural or seminatural fragments of vegetation^1–5^. The adaptive response to such drastic changes varies widely among species. Susceptible species experience isolation and population decline, and may become locally extinct^1,6,7^. For some species (“urban avoiders”), buildings, streets, highways, and other human settlements restrict their movement, reducing the connection within the metapopulation^8–10^. These barriers are expected to reduce gene flow and genetic diversity, increasing inbreeding, allele fixation and genetic structure of those species trapped in isolated vegetation fragments^11^. However, contrasting results are expected for those species for which population size and connectivity increase in urban areas (“urban exploiters”)^9,12^.

The effect of urbanization on gene flow and other population genetic parameters will depend on each species’ mobility and life history traits^13^. In birds, a species tolerance and adaptability to urban environments determines the impact of negative effects from habitat fragmentation and isolation^12^. In wrentits (*Chamaea fasciata*), a species with low dispersion capability, major roads restricted gene flow between fragments in urban landscape, producing a strong genetic structure across populations^14^. Similarly, in a species with high dispersion capability, as the song sparrow (*Melospiza melodia*), urban land cover has also shaped its genetic structure^15^. In this case, higher genetic structure occurred in sites isolated and surrounded by dense and old urban development, compared to more recently developed urban sites or even between forest relics^15^. However, in low-density urbanized sites, song sparrows showed no evidence of population genetic structure, suggesting that this species is capable of maintaining sufficient gene-flow between sites in non-saturated urban landscapes^16^. In great tits (*Parus major*), differences in patch size, population size and fragmentation may have different effects over genetic structure levels^12,17,18^. The distance between patches within an urban matrix has reduced connectivity between patches, and increased the genetic structure of the Singaporean yellow-breasted babbler (*Mixornis gularis*), a tropical edge-tolerant species^19^. These examples suggest that the effects of urbanization on structuring population genetics vary within and between species.

It is possible to use different approaches to evaluate the effects of urbanization on gene flow and genetic structure. Methods based on circuit-theory are well fitted to evaluate the effect of the complex connectivity structure within urban habitats on the genetic structure of mobile individuals such as birds^20^. This method focuses on random walking and multiple-choice path algorithms to uncover current flow in a resistance matrix developed from geographic information systems (GIS)^20^. Circuitscape models the probability that individuals disperse or move between populations depending on the permeability of the landscape. With this algorithm landscape resistance hypotheses can be proposed, assigning different resistance values to each cell, or portion of the landscape such as forest cover, altitude or topological characteristics; based on each species natural history and habitat use. Values of cumulative resistance among populations may be correlated with genetic structure^15^.

In this study, our main objective was to evaluate the effect of urbanization on genetic diversity and gene flow patterns of two neotropical bird species with different levels of resilience to urbanization, across a heterogeneous landscape of urbanization and agriculture in Costa Rica. The white-eared ground-sparrow (*Melozone leucotis*) inhabit thickets, coffee plantations and secondary forest edges^21–23^. Historically, coffee plantations expanded in the early twentieth century, and presumably provided greater connectivity for white-eared ground-sparrow; but in recent years, this habitat type has been gradually transformed into urban settlements and the remaining habitat of the white-eared ground-sparrows has been converted into fragments that vary in isolation^1,24–26^. On the contrary, the second species included in this study, the house wren (*Troglodytes aedon*), is commonly found in open and semi-open areas, forest edges and it has presumably benefited from urban development^21,27,28^. Their territories are smaller in more urbanized areas, and this has been associated with an increase in nesting and food resources in these areas^27^. To date, there is a paucity of studies that evaluate the impact of recent habitat fragmentation and habitat loss on genetic diversity and structure of tropical species that differ in their response to the changes imposed by urbanization*.*

## Results

One marker for each species was monomorphic (*Asµ18* and *ThPl-01*) across all populations, so we removed them from further analyses. For the white-eared ground-sparrow, only *Mme7* deviated from Hardy Weinberg Equilibrium (HWE) as expected for a sex-linked marker. To include it in further analysis, the missing allele was coded as missing for females and juveniles^15^. Locus heterozygosity (*H*_*o*_) ranged between 0.347 and 0.903 and *H*_*e*_ ranged 0.368–0.891 for white-eared ground-sparrows (Supplementary Table S1). In house wrens, locus *H*_*o*_ ranged between 0.107–0.904, while *H*_*e*_ ranged from 0.101 to 0.870 (Supplementary Table S1). Overall, *H*_*e*_ was consistently higher for white-eared ground-sparrows (0.639, 95% CI = 0.510 and 0.785) than for house wrens (0.519, 95% CI = 0.327 and 0.711), though confidence intervals of diversity estimates overlapped between species. Inbreeding was not observed in any species, but we found a heterozygote excess in the white-eared ground-sparrow in the UCR subpopulation (Table 1).

**Table 1:** Mean (± SD) observed heterozygosity (H_O_), expected heterozygosity (H_e_), inbreeding coefficient (F_IS_) and allelic richness (A_r_) for each population assessed for white-eared ground-sparrow (*Melozone leucotis*) and house wren (*Troglodytes aedon*).

Species	Population	n	H_O_	H_e_	F_IS_	A_r_
*Melozone leucotis*	MTV	12	0.655 ± 0.243	0.640 ± 0.256	− 0.048 ± 0.139	5.86
	HDA	12	0.667 ± 0.180	0.620 ± 0.222	− 0.104 ± 0.127	5.57
	UCR	13	0.615 ± 0.335	0.518 ± 0.309	− 0.215 ± 0.127	4.94
	JBL	19	0.597 ± 0.223	0.570 ± 0.207	− 0.057 ± 0.186	4.54
*Troglodytes aedon*	MTV	11	0.468 ± 0.321	0.489 ± 0.273	0.025 ± 0.297	3.76
	HDA	11	0.416 ± 0.310	0.468 ± 0.297	0.096 ± 0.308	4.37
	UCR	16	0.411 ± 0.318	0.483 ± 0.317	0.140 ± 0.259	4.08
	JBL	13	0.517 ± 0.287	0.522 ± 0.283	− 0.017 ± 0.183	4.45
	COR	10	0.500 ± 0.245	0.460 ± 0.226	− 0.095 ± 0.214	3.00

We found significant genetic structure in both species, *θ*_*WC*_ = 0.0872, (95% CI = 0.0463 and 0.1411) for the white-eared ground-sparrow, and *θ*_*WC*_ = 0.0610 (95% CI = 0.0422 and 0.0736) for the house wren. Pairwise F_ST_ differed significantly between all but one comparison (UCR and HDA) for white-eared ground-sparrows (Supplementary Table S2). For house wrens, only MTV and COR had significant differences in allele frequencies from all the other populations, while UCR and JBL marginally differ between each other (Supplementary Table S2).

**Figure 1:**
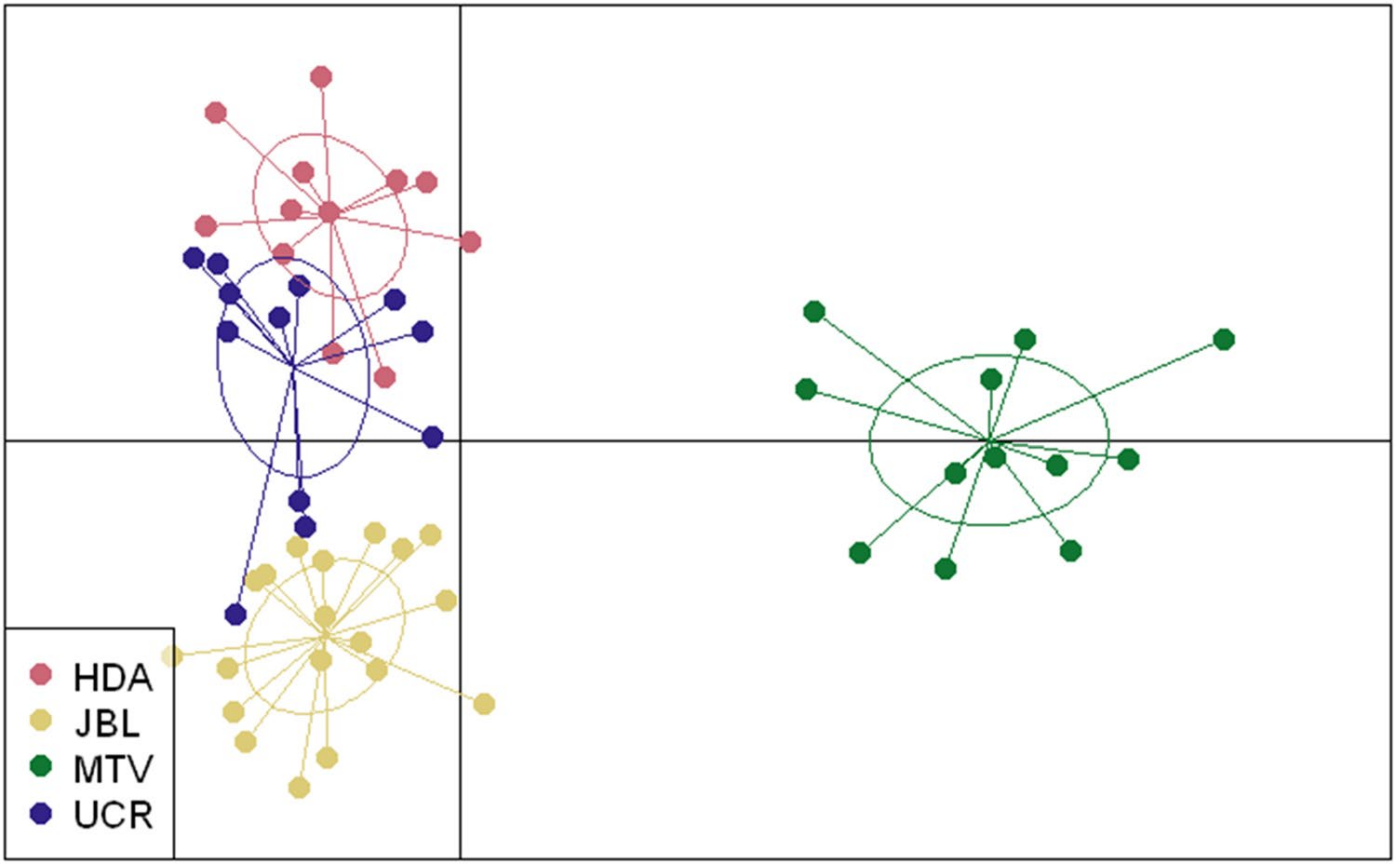
DAPC clustering for individuals of white-eared ground-sparrows (*Melozone leucotis*) for Heredia (HDA), Lankester Botanical Garden (JBL), Monteverde (MTV) and University of Costa Rica (UCR) populations with priors set as sampling sites, retaining 27PC after cross-validation.

**Figure 2:**
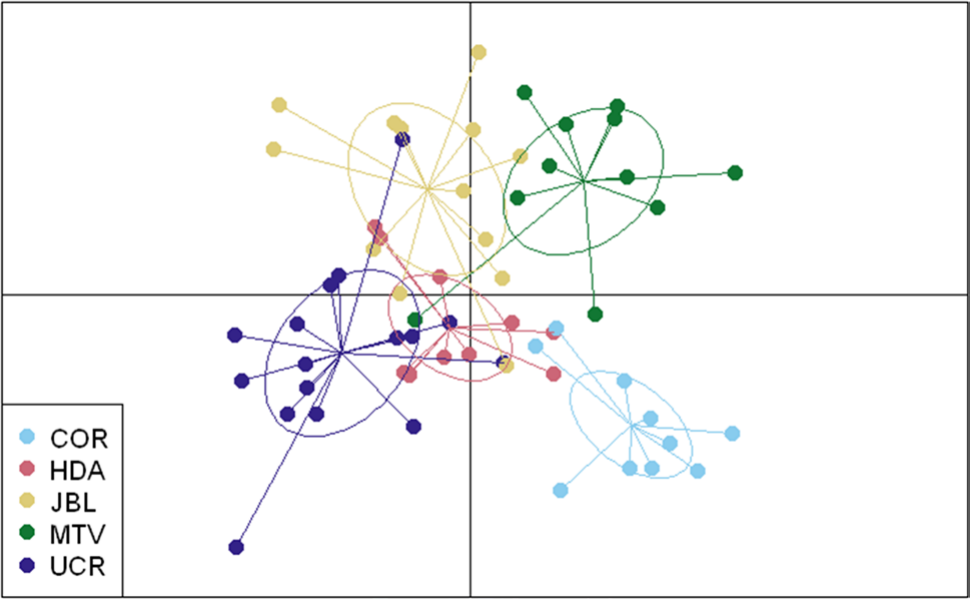
DAPC clustering for individuals of house wrens (*Troglodytes aedon*) for Heredia (HDA), Lankester Botanical Garden (JBL), Monteverde (MTV), University of Costa Rica (UCR) and Corredores (COR) populations with priors set as sampling sites, retaining 18PC after cross-validation.

**Figure 3:**
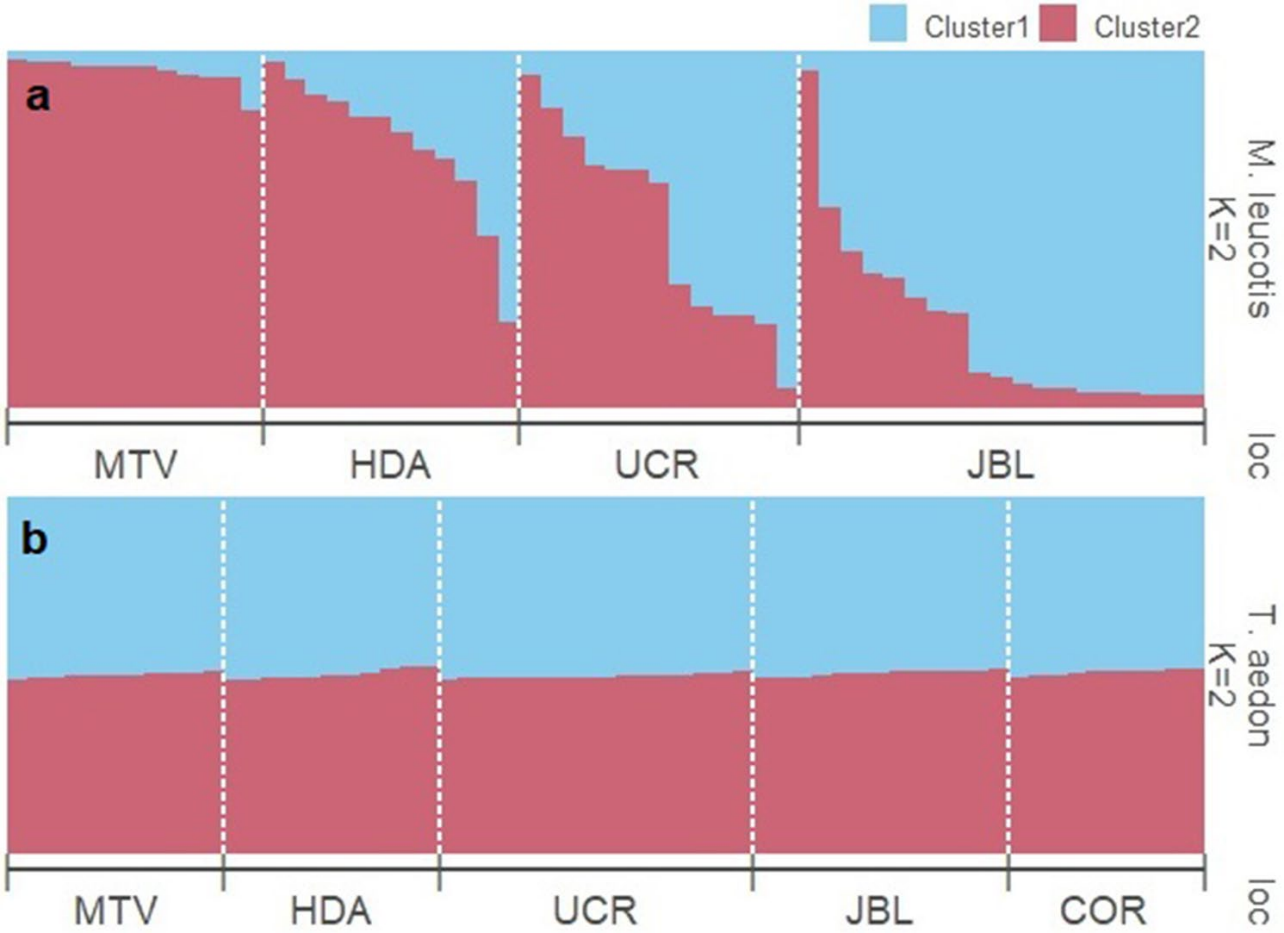
STRUCTURE barplots for (**a**) White-eared ground-sparrow (*Melozone leucotis*) *K* = 2, and (**b**) House wren (*Troglodytes aedon*) *K* = 4. Plots are sorted by sampling location, bars are sorted by Cluster 1 proportion and each bar represents one individual, while colors represent each cluster, and its size corresponds to assignment proportion. *MTV* Monteverde, *HDA* Heredia, *UCR* University of Costa Rica, *JBL* Lankester Botanical Garden, *COR* Corredores.

In white-eared ground-sparrows MTV clustered separately from the rest of the populations, HDA and UCR grouped together and JBL separated from HDA and UCR on the second axis (Fig. 1), using DAPC clustering with priors defined as sampling sites. In contrast, for the house wren, UCR, HDA, JBL and MTV clustered closely together, while individuals from COR were more separated from the other populations (Fig. 2). To evaluate the possible effect of COR affecting the similarity of the other house wren populations in the DAPC analysis, we performed a second DAPC analysis excluding this population, and obtained similar results; all populations clustered closely together (Supplementary Fig. S1). For the white-eared ground-sparrow, the Evanno method grouped individuals into *K* = 2 clusters (Supplementary Fig. S2). With *K* = 2, all MTV individuals were assigned to Cluster 1, while HDA, UCR and JBL individuals represented a mixture from both clusters (Fig. 3a). For house wrens, the Evanno method indicated that *K* = 2 was the most likely configuration, but the highest L(*K*) likelihood corresponds to *K* = 1 (Supplementary Fig. S2). Individuals from each population of house wrens were equally assigned to both clusters, suggesting a single panmictic population (Fig. 3b).

**Figure 4:**
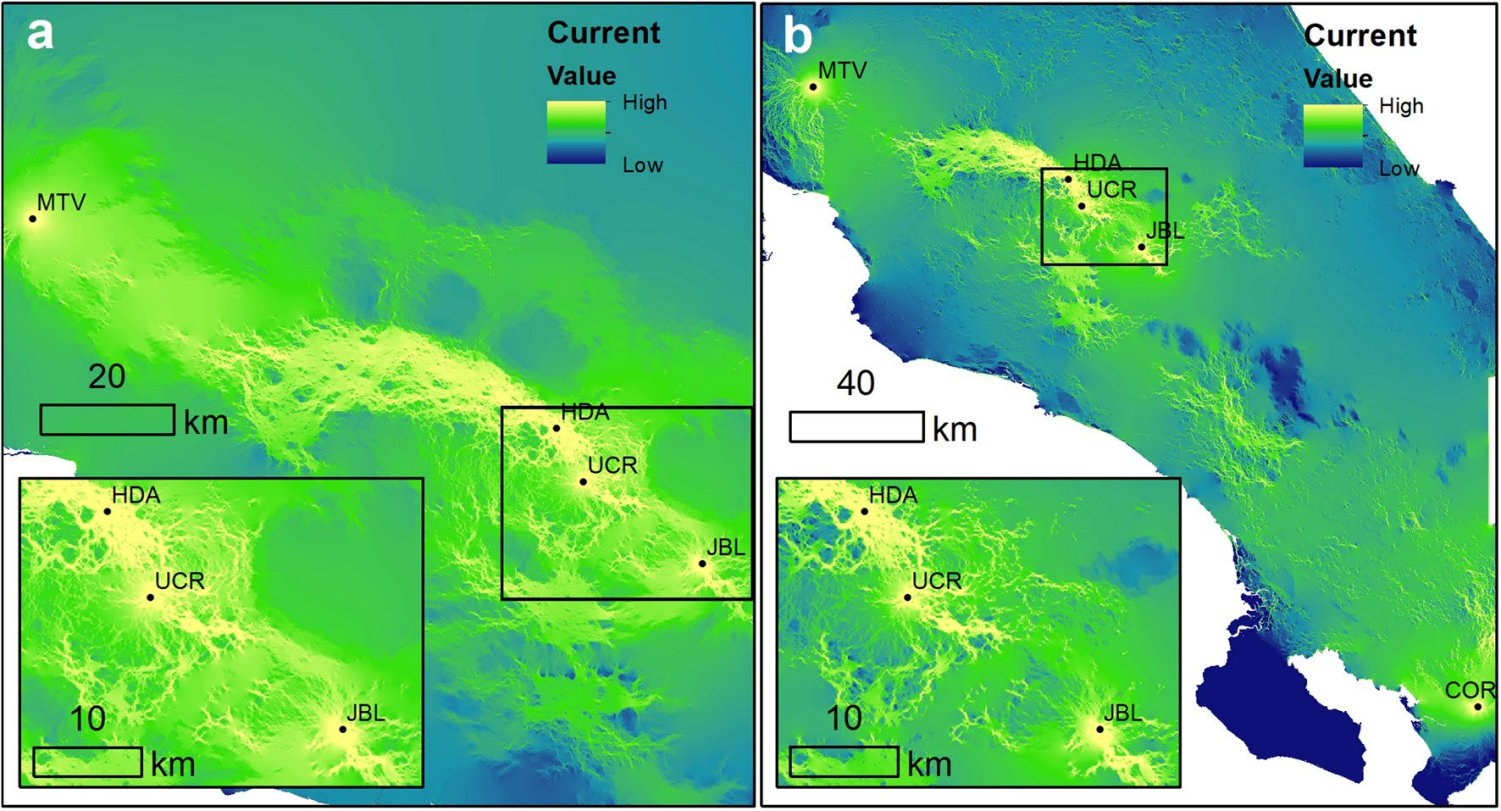
Maps of cumulative current within sampling site, modelled with circuit theory algorithm using Circuitscape 4.0, based on a land coverage layer of Costa Rica in 2005. Resistance values for each category are described on Supplementary Table S4. (**a**) corresponds to white-eared ground-sparrow (*Melozone leucotis*) sampling sites, and (**b**) house wren (*Troglodytes aedon*) sampling sites. *MTV* Monteverde, *HDA* Heredia, *UCR* University of Costa Rica, *JBL* Lankester Botanical Garden, *COR* Corredores.

We found significant IBD for white-eared ground-sparrows (r = 0.469, p < 0.001, Supplementary Fig. S3a) but not for the house wren (r = 0.066, p = 0.184, Supplementary Fig. S3b). The urban resistance circuit analysis showed high connectivity between UCR and HDA, and lower connectivity among JBL and both HDA and UCR, for both species (Fig. 4). Connectivity between MTV and all other populations was low; a similar result was found for COR in the house wren (Fig. 4a,b). We found that the urban resistance matrixes correlated with genetic differences for both species, but correlations were higher for white-eared ground-sparrow (white-eared ground-sparrow: r = 0.350, p < 0.001; house wren: r = 0.114, p = 0.020).

**Figure 5:**
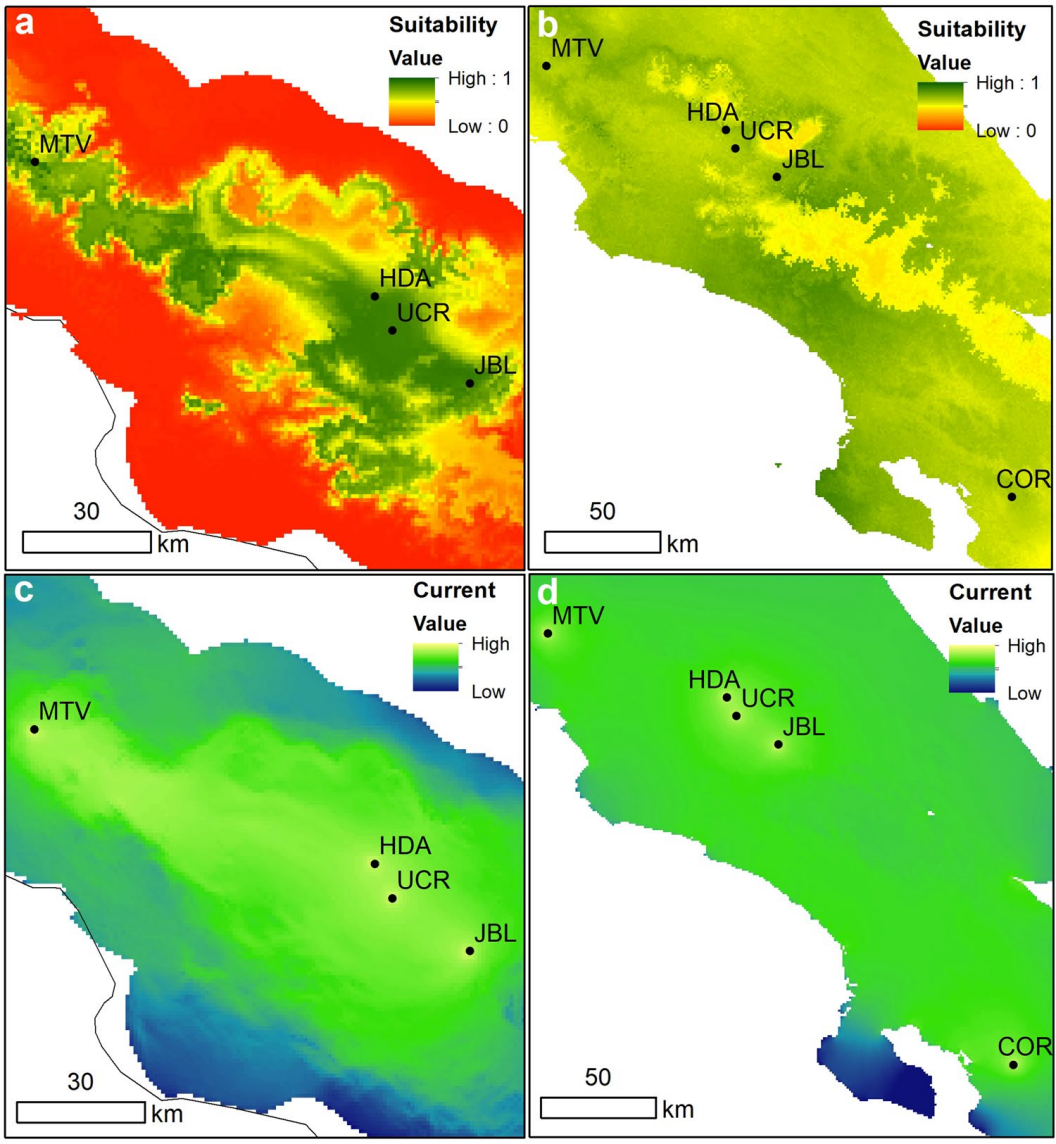
Maps of (**a**,**b**) habitat climatic suitability using MAXENT with clog-log transformation for (**a**)**.** white-eared ground-sparrow (*Melozone leucotis*) and (**b**) house wren (*Troglodytes aedon*) in Costa Rica. (**c**,**d**) Represents maps of cumulative current within sampling site, modelled with circuit theory algorithm using Circuitscape 4.0, based on MAXENT climatic suitability prediction for (**c**) white-eared ground-sparrow sampling sites and (**d**) house wren sampling sites*.* *MTV* Monteverde, *HDA* Heredia, *UCR* University of Costa Rica, *JBL* Lankester Botanical Garden, *COR* Corredores.

Our niche modelling predicted that white-eared ground-sparrows should be restricted to the forested areas between the central valley of Costa Rica and MTV (Fig. 5a). For house wrens, we found a countrywide suitability (Fig. 5b). Climatic resistance values correlated with genetic Euclidean distances for both species, but with a higher correlation for white-eared ground-sparrows (r = 0.453, p < 0.001; Fig. 5c) than for house wren (r = 0.100, p = 0.034; Fig. 5d).

## Discussion

We found that urbanization differently affected gene flow levels of each species. Urbanized areas do not represent a barrier for house wrens, consequently this species appears to be a single panmictic population in Costa Rica. On the contrary, gene flow is restricted by urban development in the white-eared ground-sparrow. Using microsatellites, our analyses showed higher genetic structuring in white-eared ground-sparrow than in house wren. STRUCTURE and DAPC suggested differences in allele frequencies among white-eared ground-sparrow populations, while clustering all house wren individuals into a single group.

Genetic diversity estimates were similar for both species because confident intervals overlapped. These results contradict our initial predictions, since we expected a higher genetic diversity for house wren which is an urban tolerant species that shows great plasticity in habitat and nesting site preferences^27–29^. On the other hand, we expected white-eared ground-sparrow to have less genetic diversity since this species is isolated in small populations that are expected to experience higher genetic drift^12,27^. Similar results were found in two European bird species. In this study both species, the tolerant blue tit (*Cyanistes caeruleus*) and less tolerant great tit*,* had similar genetic diversity levels in an urban area, even though genetic structure was significantly higher for the great tit than for the blue tit^30^. Other studies have reported similar genetic diversity estimates between urban and rural populations of the same bird species (e.g., song sparrow; burrowing owl, *Athene cunicularia*; house sparrow, *Passer domesticus*)^16,31,32^. Therefore, our results may be explained by large effective population sizes in both species. The recent urbanization of our study area (< 60 years)^33^, has not allowed sufficient time to pass to reduce population sizes and consequently genetic diversity through drift. This is emphasized by the fact that that habitat fragmentation has occurred gradually^33^, which probably allowed remnant forest patches to act as stepping-stones for gene flow in the last three decades^1,24,34^.

Clustering algorithms suggest that urbanization imposes a barrier to gene flow in white-eared ground-sparrow, genetically structuring its populations. In contrast, sampled populations in our study of the house wren in Costa Rica may be considered a large panmictic population. Habitat loss and fragmentation often limit connectivity and gene flow and, consequently, increase differences in allele frequencies between populations^35^. Multiple studies have shown how urban areas increase fragmentation and isolation of avian populations with its corresponding effects on genetic diversity^14,15,18,19,30^. However, the magnitude of the effects of urbanization varies among species. For example, differences in sensitivity to urbanization resulted in higher genetic structure for great tits than for blue tits in Poland, in which fragmentation (urban *vs.* forest) and isolation by urban areas, limited gene flow for great tits but did not for blue tits^30^. The authors attribute these differences to the greater migration and dispersal ability of blue tits in urban populations^30^. Tan et al*.*^19^ also found that the effective population size of the forest-edge tolerant Singaporean yellow-breasted babbler, correlates with the habitat contraction caused by urbanization. For the babbler, genetic differences in urban subpopulations were attributed to small effective population sizes, limited dispersal, and a lack of connectivity between patches^19^. The differences we observed on genetic structure between white-eared ground-sparrow and house wren may also be explained by contrasting tolerance to urbanization between these two species^27^. White-eared ground-sparrows and house wrens differ in how they can benefit from urban habitats. White-eared ground-sparrows feeds in the humid leaf litter and requires thickets to build nests, while house wrens feeds on arthropods on a wide array of substrates and can build nests in buildings and human made cavities^22,27–29^. A large proportion of the original habitat occupied by white-eared ground-sparrows in Costa Rica is now located within the Greater Metropolitan Area (GAM), characterized by a recent, rapid, and unplanned urban development^36^. For the earlier half of the twentieth century, a large portion of the GAM was covered by coffee plantations and second growth vegetation, but recent urban development has reduced coffee plantations and natural vegetation cover to small, isolated fragments^1,26^. Hence, the preferred habitat of the white-eared ground-sparrow has been constantly reduced; threatening the viability of this species’ populations^25,37,38^.

House wrens inhabits a wider array of habitats and can move between populations across large, urbanized areas^29^. Accordingly, we were not able to find evidence of isolation by distance or by habitat resistance. However, we found intermediate levels of genetic structure likely attributed to its low natal dispersal, because young individuals do not need to move long distances to find suitable habitat^39^. However, this hypothesis does not explain why we did not find significant IBD. This issue will be discussed below in further detail.

From the four populations of white-eared ground-sparrows, two (HDA and UCR, Fig. 1) were clustered together. These two populations are separated by the shortest distance and likely interconnected through remnant coffee plantations and patches of second growth vegetation. This suggests that gene flow for white-eared ground-sparrows is dispersal limited possibly due to urban areas limiting migration among populations^27^. JBL is separated from HDA and UCR by a large, urbanized area of approximately 2280 ha^40^, and by a mountainous densely forested terrain which likely limited migration and gene flow. These results are supported by the high correlation between urban resistance and genetic differentiation for the white-eared ground-sparrow. MTV is geographically the most distant site, but clustered closer to HDA and UCR, due to large patches of coffee plantations located among these sites^37^. Contrary to white-eared ground-sparrow, our results suggest that house wrens may be a single panmictic metapopulation. This is supported by the lack of IBD, and the weak correlations between genetic differentiation with urban resistance and climatic resistance. These results support our initial expectations that this species benefits from the recent urban development which increases the availability of nesting sites and habitat use, thus increasing connectivity among populations^27,29^. In contrast we expect gene flow to decrease for white-eared ground-sparrow in future generations. This is expected for city populations of an urban avoider that may reach the lowest densities in urban areas^9,27^.

All our isolation models suggest that geographic distance, urban resistance, and climatic resistance limit gene flow for white-eared ground-sparrows. Distance accounted for the strongest effect on genetic structure, which suggests that this species has limited dispersal, probably related to its territoriality^41,42^, low natal dispersal, and philopatry, though these factors remain to be tested. However, evidence suggest that house wrens also have low natal dispersal and philopatry, which supports our initial assessment that differences in genetic structure between white-eared ground-sparrows and house wrens may be predominantly attributed to urbanization^39,43^. The geographic distribution of white-eared ground-sparrows in Costa Rica, which coincides with the largest urbanized area, further limits movement between populations as indicated by the high correlation between the resistance matrix and genetic structure in our estimates^23,44,45^. Another study using circuit theory analysis showed a similar effect from urbanization on population differentiation in song sparrow populations, concluding that urbanization reduces gene flow in a mobile, disturb-tolerant species^15^. Other studies have drawn similar conclusions, finding that habitat fragmentation caused by urbanization expansion affects gene flow among isolated populations, particularly for species with a limited adaptive response to such drastic changes^14,15,30^.

In contrast, genetic structure did not correlate with distance in the house wren, though genetic structure correlates weakly with environmental resistance. These results may be related to its widespread distribution and tolerance to changes imposed by urbanization. The house wren is a generalist species that nests in different man-made cavities and feeds on arthropods from a large variety of substrates^28,29^. However, 20 years ago, a study of six house wren populations in Costa Rica found significant IBD and genetic structure^39^. Differences between both studies may be attributed to the rapid urban expansion that occurred in the last 20 years in Costa Rica, which likely has increased dispersal and gene flow in the house wren. Additionally, most populations studied by Arguedas & Parker^39^ at that time were separated by forested areas, which is a barrier for dispersion in this species, producing less connected populations.

Our results provide evidence for the need for conservation strategies and management for non-resilient species whose habitat has been reduced to small fragments immersed in urban matrices. The neotropics are the most avian diverse region, but the rapid growth of overcrowded cities poses a risk for urban avoider birds^1,46,47^. This is the case of white-eared ground-sparrow that may struggle to withstand the effects of the uncontrolled sprawl of Costa Rican urbanization^36^. Urban expansion will further isolate white-eared ground-sparrow populations and reduce their available habitat, increasing the effect of drift and inbreeding, which may increase the risk of local extinction^48,49^. Other species with similar ecology and behavior than white-eared ground-sparrow may be exposed to the same consequences of urbanization. It is then urgent to implement management and conservation strategies that ameliorate the negative effects of urbanization expansion on the rich Costa Rican avifauna^1^.

In conclusion, our study shows that the effect of unplanned urbanization sprawl on genetic diversity may strongly depend on a species ecology and behavior. Our results indicate that urbanization limits gene flow and has an effect in the genetic structure of white-eared ground-sparrows, but barely affects house wrens. Such differences are associated with differences in tolerance to urban ambient and the resources that these landscapes offer to each species. White-eared ground-sparrow is more sensitive to urbanization and habitat loss than house wren, which may result in lower diversity if habitat availability is further reduced. We are aware that our sample sizes may limit our conclusions, however, differences between species were consistent across all our analyses and our results contribute to the scant information on the vulnerability of city avoider species, such as white-eared ground-sparrows. Studies on gene flow in neotropical cities are fundamental to implement conservation strategies that increase connection between patches of natural vegetation immersed in extensive urban matrices.

## Methods

### Study site

**Figure 6:**
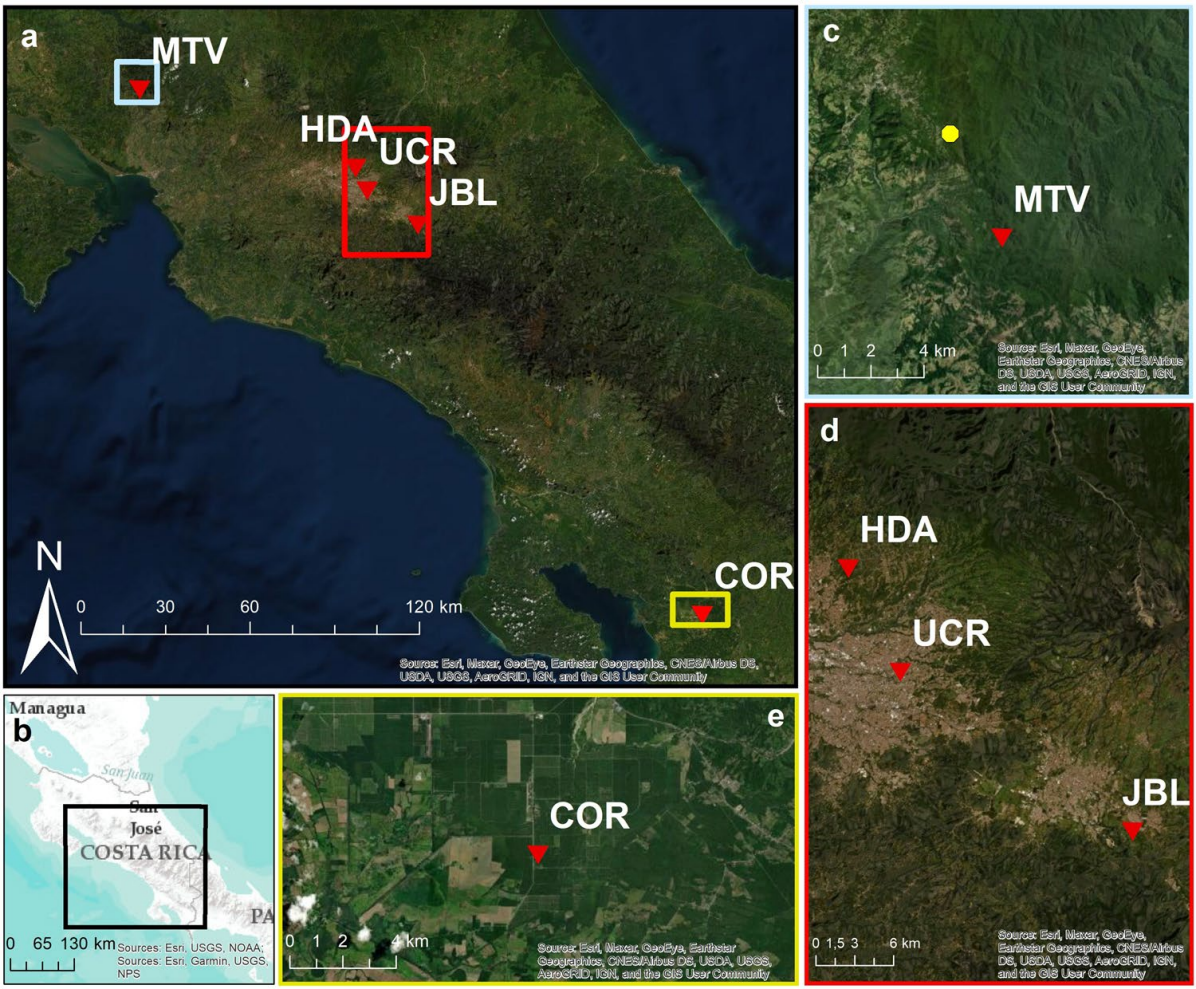
Satellite images of the sampled locations (red down triangle). (**a**) Satellite map showing all sampling sites. (**b**) Our study was located in Costa Rica, Central America. (**c**) MTV sampling site. Yellow dot corresponds to the MTV sampling location for white-eared ground-sparrow. (**d**) HDA, UCR, and JBL sampling sites, located within the Great Metropolitan Area. (**e**) COR sampling site. *MTV* Monteverde, *HDA* Heredia, *UCR* University of Costa Rica, *JBL* Lankester Botanical Garden, *COR* Corredores. Images base map source: Esri, Maxar, GeoEye, Earthstar Geographics, CNES/Airbus DS, USDA, USGS, AeroGRID, IGN, Garmin, NOAA, NPS, and the GIS User Community.

We selected four sites for white-eared ground-sparrow (Fig. 6), three located within the Greater Metropolitan Area of Costa Rica (GAM). This is the most densely populated and urbanized area of the country, and though some remaining coffee, sugarcane, and other plantations are still present; most of the area is urbanized. We sampled individuals in Heredia province (**HDA**: 10° 01ʹ N, 84° 05ʹ W, altitude: 1200–1500 m); University of Costa Rica Campus, San José Province (**UCR**: 09° 56ʹ N, 84° 05ʹ W, altitude: 1200 m); and Lankester Botanical Garden, Cartago Province (**JBL**: 09° 50ʹ N, 83° 53ʹ W, altitude: 1400 m). Both Lankester Botanical Garden and University of Costa Rica Campus are urban areas with small patches of secondary vegetation immersed in an urban matrix. HDA had a lower urban coverage, surrounded by both urban zones and coffee plantations. The other sampling site was Monteverde, in the Puntarenas province (**MTV**: 10° 18ʹ N, 84° 48ʹ W; altitude:1600 m), characterized by low density population, coffee plantations, and large forest patches that connect with more extensive mature forests.

For house wren*,* we collected individuals in five localities (Fig. 6); three of these localities were the same sampling sites of white-eared ground-sparrow: HDA, UCR and JBL. The MTV sampling site of house wren was located about 5 km south of the white-eared ground-sparrow sampling site (10° 16ʹ N, 84° 47ʹ W, altitude: 1090 m). The fifth sampling site was in Corredores, in the Puntarenas Province (**COR**: 08° 35ʹ N, 82° 58ʹ W; elevation 11 m), and is characterized by extensive oil palm plantations.

### Sample collection

We captured individuals of white-eared ground-sparrow using mist nets upon song playback in 2012, 2013, and 2019–2021, capturing between 12 to 19 individuals per site. House wren individuals were captured using mist nets upon song playback in 2019–2021, capturing between 11 and 16 individuals per site. We color banded all collected individuals, sexed all adults, and categorized juveniles as indeterminate. We collected 10–30 µL of blood from the brachial vein for each individual and stored it in 95% ethanol or Lysis buffer for molecular analyses^50^. Anesthesia was not used, and all birds were released unharmed. Procedures were conducted in accordance with the current laws in Costa Rica. Research permits and animal handling protocols were approved by the Research Committee of Biology School and by the Animal Care Committee (ACC) of Universidad de Costa Rica under the project approval code B9-469 and C1-085 of Vicerrectoría de Investigación. All procedures were conducted following the regulations of the ACC.

### DNA extraction and SSR amplification

We extracted DNA from blood and muscle tissue using the DNeasy blood and tissue Kit (Qiagen Inc., Valencia, CA, USA). For white-eared ground-sparrow*,* we amplified eight primers: *Mme2*, *Mme7*, *Mme8*, *Asµ15*, *Asµ18*, *Escµ6*, *Gf01* and *Gf05*^51–54^. For house wren, we amplified eight primers: *ThPl-01*, *ThPl-14*, *ThPl-17*, *Ta-C6-7*, *Ta-B4-2*, *Ta-A5-2*, *Ta-A5-15* and *Ta-C3(B)-2*^55,56^. Forward primer of each pair was 5’ fluorescently labelled. PCR thermal profiles and primer mixes are described in Supplementary Table S3. We amplified all markers in a 12.5µL reaction containing 0.4 µM primer mix and 40 ng of template DNA. We used Multiplex Master Kit (Qiagen) for mixed primers and Top Taq Master Kit (Qiagen) for *Gf01*. We used Veriti™ thermal cycler (Applied Biosystems, Foster City, CA, USA) to perform the PCR reaction. Capillary electrophoresis was done in a 3500 Genetic Analyzer (Applied Biosystems) at Escuela de Biología, UCR, using Hi-Di™ Formamide and GeneScan™ 500 LIZ™ dye Size Standard (Applied Biosystems). We scored genotypes using GeneMarker 1.91 (SoftGenetics, State College, PA, USA).

### Genetic analysis

We conducted population genetics analyses in R 4.0.2^57^, using the RStudio 1.3.180 GUI^58^. We calculated expected heterozygosity (H_e_), observed heterozygosity (H_o_), inbreeding coefficients (F_IS_) and allelic richness (A_r_) per population (to compensate for differences in sampling size per population) using the “hierfstat” package^59^. We calculated Weir and Cockerham F_ST_ (*θ*_*WC*_)^60^, expected heterozygosity values and 95% confidence interval of both estimates using 9999 bootstrap with *boot.vc* function of the “hierfstat” package. We also calculated pairwise *θ*_*WC*_ between populations with *pairwise.WCfst* function and it’s 95%CI using *boot.ppfst* by 9999 bootstrap among loci using the “hierfstat” package.

We visualized individuals' similarity using a discriminant analysis of principal components (DAPC) as implemented in the “adegenet” package^61^. To determine the most suitable number of PC axis to include in the analysis, we performed cross-validation using the *xvalDapc* function, with 30 repetitions. We then narrowed the search window to the nearest six PC surrounding the previous selected PC while increasing replicates to 999. The Bayesian clustering methods in Structure 2.3.4 allowed us to assess the possible genetic clustering of individuals into a *K* number of clusters^62^. The admixture model and correlated allele frequencies were used, with 300,000 markov chains and a burn-in of 30 000. Phase information was added to include *Mme7* marker in the analysis, since previous evidence has shown this is a Z-linked marker^15,53^. We tested 1 to 6 clusters with 20 repetitions for each cluster. We determined the most likely number of K clusters using Structure Harvester 0.6.94^63^ and the Evanno^64^ and Ln(K) methods. Then, we conducted another structure analysis after determining the most likely K, increasing generations and the burn-in to 1 000 000 and 100 000, respectively.

### Isolation by distance, isolation by resistance and isolation by environment

To test for isolation by distance (IBD) we estimated the correlation between pairwise Euclidean genetic distances and geographic linear distances among individuals using a Mantel test after 9 999 permutations. The same coordinates of the sampling sites were assigned to all individuals within each population.

We estimated landscape resistance to movement and isolation by resistance (IBR) using Circuitscape 4.0.5^65^. To create the resistance raster, we used a 30 m resolution land coverage of 2005 shapefile layer of continental Costa Rica created by the Earth Observatory Systems Laboratory of the University of Alberta, based on Landscape 7 satellite image classification and interpretation into 16 categories (Supplementary Table S4), part of *Atlas Digital de Costa Rica* 2014 project^66^. We also used a 30 m resolution altitude layer developed by the Atlas Digital de Costa Rica 2008 project raster^66^. To create a resistance grid, we rasterized the land cover into a 100 m grid and classified the altitude raster into 200 m elevation categories using ArcGis 10.6 and ArcGis toolbox “Gnarly Landscape Utilities”^67^, based on resistance values assigned to each land use and altitude categories, according to expert opinion on habitat suitability for both species (Supplementary Table S4). To assess the effect of urbanization on the gene flow of both species, we assigned highest resistance to the urban land cover for both species, based on the assumption that urbanization impacts both species in the same way. As altitude and land use layers overlap, the maximum value of resistance between both layers was assigned to each grid. We performed a pairwise circuit theory analysis of connectivity between all sampling locations of white-eared ground-sparrow and house wren. Pairwise calculation is an iterative algorithm that allows to obtain the connectivity and resistance between each pair of focal nodes, in which one will be assigned a current source of 1 A and the other will be assigned as ground connection. We performed a log_10_ transformation for the cumulative current map for easier visualization. Finally, the correlation between pairwise cumulative resistance values and genetic differentiation among individuals was also tested using a Mantel test with 9999 permutations.

To test for isolation by environment (IBE), we developed a maxent species distribution model using the “Wallace” R library^68^. We obtained the occurrence of each species from the eBird database (www.ebird.org). We then removed duplicated data, erroneous observations, and randomly thinned spatial data to 1 km for white-eared ground-sparrow and 5 km for house wren. We used all 19 bioclimatic data available at www.worldclim.org at a 1 km^2^ resolution. We delimited the area of calibration to 25 km radius around each occurrence point. We sampled 10 000 background points and used the spatially block partition method. We set regularization multiplier parameters from 0.5 to 4, with 0.5 increments and used 5 feature class combinations (“L”, “H”, “LQ”, “LQH”, “LQHP”, where “L” = linear, “H” = hinge, “Q” = quadratic, “P” = product). We evaluated the models using the area under the curve (AUC) and average 10th percentile omission rate. To convert it into a resistance matrix input for Circuitscape, we performed a *cloglog* map prediction and set it as conductance in our Circuitscape model. We developed pairwise modeling between all sampling locations of white-eared ground-sparrow and house wren with a log_10_ transformation for the cumulative current map. We performed a Mantel test with 9999 permutations between individual genetic Euclidean distance and resistance values.

## Supplementary Information

Supplementary Information.

## Data Availability

The datasets generated during the current study are available from the corresponding author upon reasonable request.
