# Supplementary material for: Genetic structure in neotropical birds with different tolerance to urbanization
Source: Sci Rep. 2022 Apr 11;12:6054. doi: 10.1038/s41598-022-09961-9 (PMC9001702; doi:10.1038/s41598-022-09961-9)
Supplement: Supplementary file 1 — Supplementary Information. [file 41598_2022_9961_MOESM1_ESM.pdf]

Genetic structure in neotropical birds with different tolerance to urbanization

Authors and affiliations:

Mauricio Rodríguez-Bardía[1,2,*], Eric J. Fuchs[2], Gilbert Barrantes[2], Ruth Madrigal-Brenes[2],Luis Sandoval[2]

1: Programa de Posgrado en Biología, Sistema de Estudios de Posgrado, Universidad de Costa Rica. San Pedro, San José, 11501-2060.

2: Escuela de Biología, Universidad de Costa Rica. San Pedro, San José, 11501-2060

***Corresponding author:** maurod.96@gmail.com, alejandro.rodriguezbardia@ucr.ac.cr

Supplementary tables S1, S2, S3, S4 and supplementary figure S1, S2, and S3.

Supplementary Table S1. Observed heterozygosity (H_o), Expected heterozygosity (H_e), inbreeding coefficient (F_{IS}) and allele number (A) for polymorphic locus used in this study for white-eared ground-sparrow (*Melozone leucotis*) and house wren (*Troglodytes aedon*).

Species	Locus	H_o	H_e	F_{IS}	A
Melozone leucotis	Mme2	0.514	0.397	-0.294	3
	Mme7†	0.601	0.530	-0.134	5
	Mme8	0.562	0.503	-0.117	4
	Asμ15	0.903	0.854	-0.057	14
	Escμ6	0.616	0.565	-0.090	5
	Gf05	0.347	0.368	0.057	7
	Gf01	0.892	0.891	-0.001	21
Troglodytes aedon	Th-Pl14	0.904	0.870	-0.039	16
	Ta-C6-7	0.384	0.550	0.302	7
	Ta-A5-15	0.351	0.335	-0.048	3
	Ta-A5-2	0.558	0.628	0.111	7
	Ta-B4-2	0.610	0.605	-0.008	9
	Th-Pl17	0.107	0.101	-0.056	2
	Ta-C3(B)-2	0.320	0.302	-0.059	5

† Z-linked locus.

Supplementary Table S2. Pairwise Weir and Cockerham FST [95% CI, 9999 bootstraps over loci] between populations for white-eared ground-sparrow (*Melozone leucotis*) (above diagonal) and house wren (*Troglodytes aedon*) (below diagonal).

	HDA	JBL	UCR	MTV
HDA		0.059 [0.008_0.100]	0.019 [-0.004_0.060]	0.065 [0.0140_0.126]
JBL	0.017 [-0.009_0.037]		0.070 [0.024_0.108]	0.161 [0.080_0.250]
UCR	0.031 [-0.017_0.077]	0.035 [0.003_0.055]		0.123 [0.014_0.255]
MTV	0.046 [0.027_0.070]	0.057 [0.023_0.099]	0.083 [0.056_0.110]	
COR	0.084 [0.057_0.111]	0.086 [0.030_0.167]	0.089 [0.038_0.125]	0.098 [0.027_0.190]

Supplementary Table S3. Primer mixes and PCR thermal conditions used for the study.

Species	Mix	Primer	Reference	Thermal profile
Melozone leucotis	1	Mme2	[1]	95 °C for 15 min; 33 cycles of 94 °C for 30 s, 58 °C for 1:30 min, 72 °C for 1:00 min; followed by 60 ° C for 30 min
		Mme7	[1]	
		Mme8	[1]	
	2	Asµ15	[2]	95 °C for 15 min; 30 cycles of 94 °C for 30 s, 55 °C for 1:30 min, 72 °C for 1:00 min; followed by 60 °C for 30 min
		Asµ18	[2]	
		Escµ6	[3]	
		Gf05	[4]	
	-	Gf01	[4]	94 °C for 3 min; 33 cycles of 94 °C for 1:00 min, 50 °C for 1:30 min, 72 °C for 1:00 min; followed by 72 °C for 10:00 min
Troglodytes aedon	3	ThPl-01	[5]	95 °C 15 min; 25 cycles of 94 °C for 30 s, 60 °C for 1:30 min, 72 °C for 1:00 min; followed by 60 °C for 30 min
		ThPl-14	[5]	
		Ta-C6-7	[6]	
	4	Ta-A5-15	[6]	
		Ta-A5-2	[6]	
		Ta-B4-2	[6]	
	5	Ta-C3(B)-2	[6]	95 °C for 15 min; touchdown 13 cycles of 94 °C for 30 s, 63-51 °C for 1:30 min decreasing 1 °C per cycle, 72 °C for 1:00 min; followed by 12 cycles of 94 °C for 1:00 min, 50 °C for 1:00 min, 72 °C for 1:00 min; followed by 60° C for 30 min
		ThPl-17	[5]	

Supplementary Table S4. Resistance value of each category of land cover and altitude designated for white-eared ground-sparrow (*Melozone leucotis*) and house wren (*Troglodytes aedon*).

Layer	Category	Resistance value for *M. leucotis*	Resistance value for *T. aedon*
Land Cover	Non Forestal	20	20
	Water	80	80
	Secondary forest	1	1
	Forest	20	20
	Mangrove swamp	100	100
	Forestry	20	20
	Non classified	100	100
	Clouds	20	20
	Agriculture	40	40
	Páramo	40	40
	Palm forest	20	20
	Limits	100	100
	Deforestation	40	40
	Coffee	1	1
	Urban use	100	100
	Burned areas	100	100
Altitude (m.a.s.l)	0-200	100	1
	200-400	100	1
	400-600	100	1
	600-800	50	1
	800-1000	1	1
	1000-1200	1	1
	1200-1400	1	1
	1400-1600	1	1
	1600-1800	1	1
	1800-2000	50	1
	2000-2200	100	1
	2200-2400	100	1
	2400-2600	100	1
	2600-2800	100	1
	2800-3000	100	50
	3000-3200	100	100
	3200-3400	100	100
	3400-3600	100	100
	3600-3818	100	100

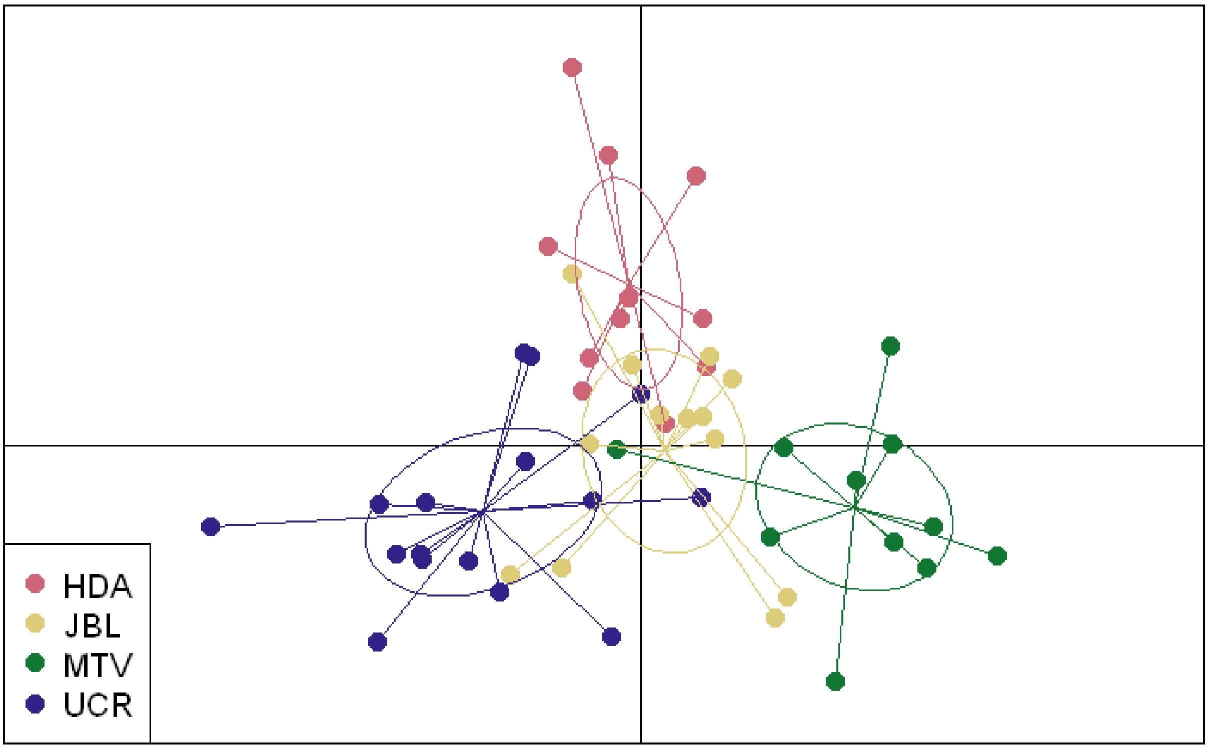

Supplementary Figure S1. DAPC clustering for individuals of house wren (*Troglodytes aedon*) for Heredia (HDA), Lankester Botanical Garden (JBL), Monteverde (MTV), and University of Costa Rica (UCR) populations, but excluding Corredores (COR) with priors set as sampling sites, retaining 15PC after cross-validation

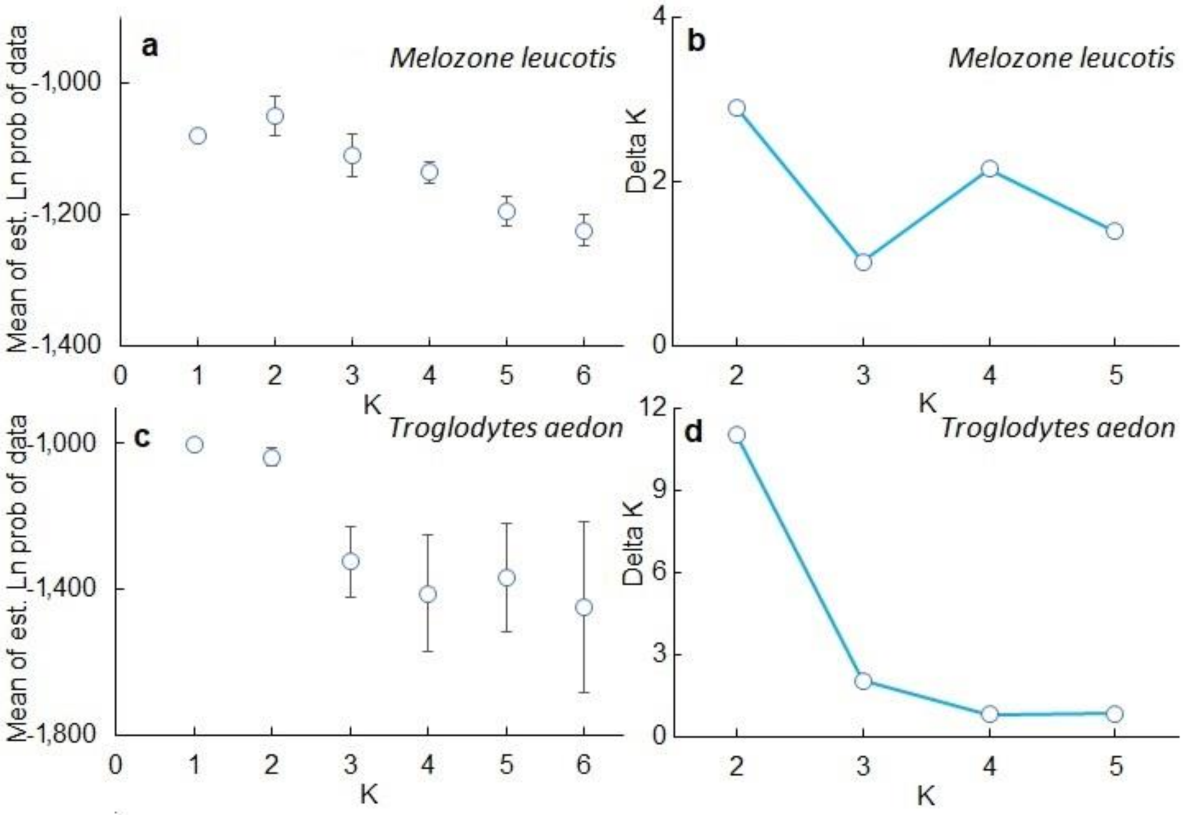

Supplementary Figure S2. Structure Harvester results: mean L(K) (a and c) and Delta K (b and d) for both white-eared ground-sparrow (*Melozone leucotis*) (a and b) and house wren (*Troglodytes aedon*) (c and d).

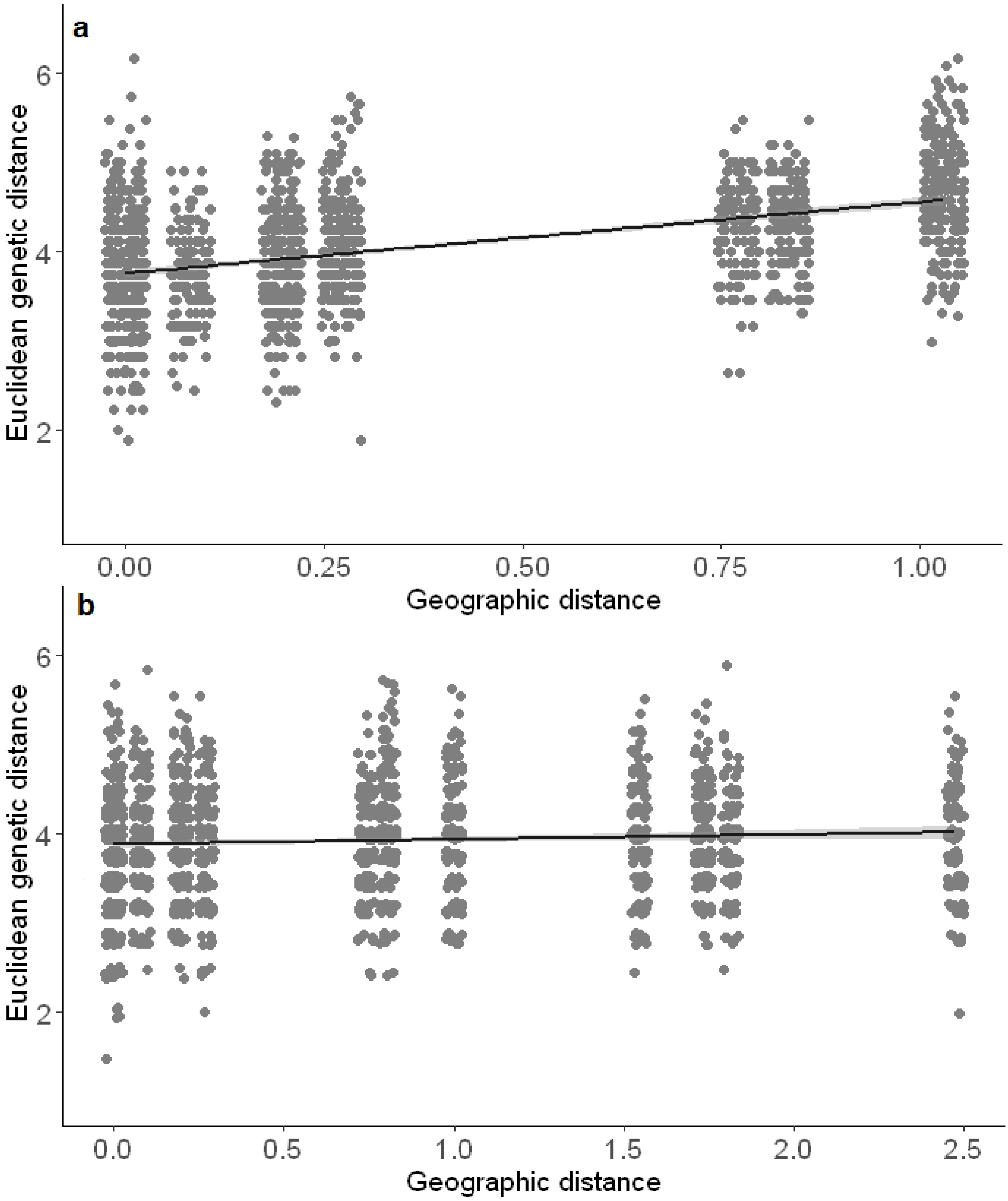

Supplementary Figure S3. Isolation by distance plot per individual with jittered points for **a.** white-eared ground-sparrow and **b.** house wren.

References

1. Jeffery, K. J., Keller, L. F., Arcese, P. & Bruford, M. W. The development of microsatellite loci in the song sparrow, *Melospiza melodia* (Aves) and genotyping errors associated with good quality DNA. *Mol. Ecol. Notes* **1**, 11–13 (2001).

2. Bulgin, N. L., Gibbs, H. L., Vickery, P. & Baker, A. J. Ancestral polymorphisms in genetic markers obscure detection of evolutionarily distinct populations in the endangered Florida grasshopper sparrow (*Ammodramus savannarum floridanus*). *Mol. Ecol.* **12**, 831–844 (2003).
3. Hanotte, O. *et al.* Isolation and characterization of microsatellite loci in a passerine bird: the reed bunting *Emberiza schoeniclus*. *Mol. Ecol.* **3**, 529–530 (1994).
4. Petren, K. Microsatellite primers from *Geospiza fortis* and cross-species amplification in Darwin's finches. *Mol. Ecol.* **7**, 1782–1784 (1998).
5. Brar, R. K. *et al.* Eleven microsatellite loci isolated from the banded wren (*Thryothorus pleurostictus*). *Mol. Ecol. Notes* **7**, 69–71 (2007).
6. Cabe, P. R. & Marshall, K. E. Microsatellite loci from the house wren (*Troglodytes aedon*). *Mol. Ecol. Notes* **1**, 155–156 (2001).
